# Reducing Inequality for Persons with Disabilities: Demystifying the ‘and’ Between Disability and Development

**DOI:** 10.1192/j.eurpsy.2023.368

**Published:** 2023-07-19

**Authors:** V. Gairola

**Affiliations:** Department of Liberal Arts, Indian Institute of Technology, Hyderabad, Noida, India

## Abstract

**Introduction:**

The aim of this paper lies in demystifying, historicizing, and de-alienating the relationship between disability and development. Both disability and development inform each other and are informed by each other in various ways which are on one hand similar and on the other hand unique. Disability is heterogeneous in nature. Disability and development are about power, access, solidarity, advocacy, inequality, rights, voice, and support. It is about accessing accessibility. It is important to understand the politics of language—how we conceptualize persons with disabilities.

**Objectives:**

The paper theorizes the ‘and’ between disability and development. What is that bridging telling us? There is already an invisible ‘and’ which joins disability and development even before this visible ‘and’ was placed in between them. It is to understand how disability is related to gender, caste, class, and poverty. The paper also looks at the government policies and adds suggestions as to what can be done practically to reduce inequality for persons with disabilities for developing a new India.

**Methods:**

This research used primary sources like books, articles, government programmes, and policies to make sense of how disability is understood and experienced in India.

**Results:**

It shows how disability and development inform each other and are informed by each other. The paper shows how each person has disabling parts and “normal” parts. Representation helps one to know the multifarious dimensions of what is awful, reprehensive, acceptable, possible, desirable, etc. Representation structures reality. Hence, they are a critical component of bringing about rights.

**Conclusions:**

It is crucial to look at the needs and challenges at the ground level contextually. It is important to understand why survival is considered sufficient and not full participation. Policies need to resonate culturally as otherwise, they tend to be confined to particular lases and groups in societies with access to technology, information, and the English language. What matters is the visibility of disability.

**Disclosure of Interest:**

None Declared

